# Effects of the copper intrauterine device versus injectable progestin contraception on pregnancy rates and method discontinuation among women attending termination of pregnancy services in South Africa: a pragmatic randomized controlled trial

**DOI:** 10.1186/s12978-016-0153-9

**Published:** 2016-04-18

**Authors:** G. Justus Hofmeyr, Mandisa Singata-Madliki, Theresa A. Lawrie, Eduardo Bergel, Marleen Temmerman

**Affiliations:** Effective Care Research Unit, Eastern Cape Department of Health/Universities of the Witwatersrand, Walter Sisulu and Fort Hare, Mthatha, Eastern Cape South Africa; Reproductive Health and Research, World Health Organization, Geneva, Switzerland; International Centre for Reproductive Health, Ghent University, Ghent, Belgium; Institute for Clinical Effectiveness and Health Policy (IECS), Buenos Aires, Argentina; Royal United Hospital, Bath, UK

**Keywords:** DMPA, IUD, Random

## Abstract

**Background:**

The copper intrauterine device (IUD) is under-utilised in South Africa, where injectable progestin contraception (IPC) dominates contraception usage. There is a lack of robust comparative data on these contraceptive options to inform policy, programs, clinical counseling, and women’s choices.

**Methods:**

Within the context of a South African program to increase women’s access to the IUD, we conducted a pragmatic, open-label, parallel-arm, randomised controlled trial of the IUD versus IPC at two South African hospitals. The target sample size was 7,000 women and the randomisation ratio was 1:1. The random sequence was computer-generated and group allocation was concealed in sealed, opaque, consecutively-numbered envelopes. Counselled, consenting women attending termination of pregnancy services were randomly assigned to IUD or IPC immediately post-termination. Condoms were promoted for the prevention of sexually-transmitted infections. The primary outcome was pregnancy; secondary outcomes were discontinuation, side-effects, and HIV acquisition and disease progression. Pregnancy and discontinuation outcomes are reported here.

**Results:**

The trial closed early with 2,493 participants randomised (IUD = 1,247, IPC = 1,246), due to international concerns regarding a possible association between IPC and HIV acquisition. Median follow-up was 20 months; 982 and 1000 participants were followed up in the IUD and IPC groups, respectively. Baseline group characteristics were comparable. Pregnancy occurred significantly less frequently among women allocated to the IUD than IPC: 56/971 (5.8 %) versus 83/992 (8.4 %), respectively; risk ratio (RR) 0.69, 95 % confidence interval (CI) 0.50 to 0.96; *P* = 0.025. There were more protocol violations in the IUD group; however, discontinuation rates were similar between IUD and IPC groups (141/855 [16.5 %] and 143/974 [14.7 %], respectively). Women in the IUD group were more likely to discontinue contraceptive use due to abdominal pain or backache and non-specific symptoms, and those in the IPC group due to oligo- or amenorhoea and lack of sexual activity.

**Conclusions:**

The IUD was significantly more effective in preventing pregnancy than IPC. Efforts to expand contraception options and improve access to the IUD in settings where it is under-utilised are worthwhile. This trial shows that randomising long-acting, reversible contraceptives is feasible.

**Trial registration:**

Pan African Clinical Trials Registry number PACTR201409000880157 (04-09-2014).

## Background

More than 4,000 terminations of pregnancy are performed each year in the East London/Mdantsanes area of South Africa and, in a survey of postnatal women attending local hospitals and clinics, two thirds of births were unintended [1]. Like most of South Africa, women utilising services at Frere and Cecelia Makiwane Hospitals and clinics have a limited choice of contraceptives and injectable progestin contraception (IPC), including depot medroxyprogesterone acetate (DMPA) and norethisterone enantate (NET), account for most contraception use. A common reason for discontinuation of contraception by women attending these services is dissatisfaction with the contraceptive method. The World Health Organization (WHO) continues to highlight the need for a wider variety of highly effective contraceptive methods to be available to women living in low and middle-income countries (LMICs) [2].

The copper intrauterine device (IUD) is a well-established, highly effective method of contraception; however, availability issues, staff capacity, provider attitude, and perception of side effects have held back its use in many LMICs, including South Africa [3–5]. In 2005, we initiated a program at Frere and Cecelia Makiwane Hospitals and clinics to expand contraceptive options to include the copper IUD (SMB® Model TCu 380A), which was previously unavailable in our services. However, we found comparative data on the relative contraceptive effectiveness of the IUD and IPC to be insufficient for adequate counselling of prospective users: Limited evidence from a 2010 Cochrane review suggested that the IUD was more effective in preventing unintended pregnancy than hormonal contraception (including IPC), but had not been further elucidated [6]; and observational data were subject to confounding as contraceptive choice often varies according to factors related to the likelihood of pregnancy. To enhance the knowledge base for our counselling and promotion of wider contraception options, we designed a pragmatic trial within our routine contraceptive services to compare effectiveness, method discontinuation, and reasons for discontinuation, of the newly introduced method (IUD) with that of the most widely-used method (IPC). As secondary outcomes, we also assessed side effects, HIV acquisition and HIV/AIDS disease progression, which we intend to report separately.

## Methods

### Study design and participants

The trial was a pragmatic, open-label, parallel arm randomised controlled trial conducted at two Eastern Cape hospitals, Frere Hospital (FH) and Cecelia Makiwane Hospital (CMH), in South Africa. Recruitment began on 6 July 2009 and ended on 8 November 2012. Women attending termination of pregnancy services at the study sites, who met the inclusion criteria and requested long-term contraception with no personal preference for a particular method, were counselled in their home language and offered participation in the trial. Women were eligible if they intended to continue contraception for at least one year, were ≥ 16 years old, had no evidence of active pelvic infection on history and clinical examination, had no contraindications to IPC or IUD use, were prepared to use either method of contraception, understood the patient information form, and were willing to sign informed consent. All women were offered counselling and voluntary HIV testing at baseline and at follow-up, according to national health policy, using rapid tests or laboratory-based ELISA tests. Those with positive results were offered a CD4 count and treatment according to national health guidelines.

### Ethics, consent, and permissions

Ethical approval for the trial was obtained from the University of the Witwatersrand Committee for Research on Human Subjects, South Africa, on the 25 April 2008, clearance certificate M080466. Signed informed consent to participate in the study was obtained from all participants.

### Randomisation

An allocation list was prepared using a computer-generated random sequence in balanced blocks of variable size and with a randomisation ratio of 1:1. Allocation slips were inserted into consecutively-numbered, opaque envelopes and sealed by a member of staff not involved in the trial. The randomisation list was sealed in a signed envelope by the same staff member for safekeeping. The randomisation envelopes were distributed to the study sites in batches of 100. Women who agreed to participate were entered onto a trial register and then enrolled by opening the next numbered, sealed envelope. The point of trial entry was at the opening of the envelope.

### Interventions

Women were fully counselled about their allocated contraception method prior to its administration. In the IUD group, the IUD was inserted after termination of pregnancy, which was usually performed by manual vacuum aspiration by the hospital staff following routine procedures. Antibiotic prophylaxis was not used. The women were offered two options regarding the string placement after full counselling about the relative benefits of each: strings protruding from the cervix by about 2 cm, or strings inserted completely within the uterine cavity or removed. The reason for offering these options is that many women in our services prefer their contraception to be confidential. In the IPC arm, women were counselled regarding the need for repeat injections at regular intervals. The first injection was administered by hospital staff according to routine practice and, in most instances, DMPA was used. However, as this was a pragmatic trial within the routine contraception services, allowance was made for the use of NET at the discretion of the provider. Women received no additional reminders to support continuation with the allocated contraception method, and no payments or other incentives were offered, other than payment of transport costs for the final visit at 12 months post-enrolment.

### Outcomes

The primary outcome was pregnancy. Secondary outcomes were method discontinuation, side effects, HIV acquisition, and HIV/AIDS disease progression. Pregnancy and discontinuation outcomes are reported in this paper.

### Data collection

Professional nurses who worked as research assistants completed a Good Clinical Practice certification course before trial commencement. Participant baseline details were entered onto a paper case report form (CRF), including demographic data, medical history, and baseline data relevant to the follow-up questionnaire (including sexual activity and potential side effects). Follow-up questionnaires were administered by the research assistants to participants by telephone at three, six, nine and 12 months after randomisation. The CRF and questionnaire were piloted before the main trial. The actual contraceptive method received after randomisation was collected retrospectively from family planning registers at the two hospitals. Participants were requested to attend an interview at the hospital 12 months after enrolment, failing which the interview was conducted by telephone. Initial and final contraceptive methods used were confirmed with the participant at the last interview. Other questions at this interview included whether the participant had been pregnant since joining the study, the outcome of the pregnancy, and what contraceptive methods had been used since joining the study, as well as reasons for discontinuation. Attempts to contact women who did not return for follow-up were continued until study closure in June 2014. Research assistants advised participants to attend the relevant health facility for any health concerns.

### Sample size and analysis

The sample size was calculated based on the primary outcome. To detect a reduction in unplanned pregnancy from 2.5 % to 1.5 %, we calculated that we needed 6,546 participants (alpha = 0.05, beta = 0.20; Epi Info™ statistical software version 7). To allow for loss to follow-up, we planned to recruit a total sample size of 7,000 women.

Intention-to-treat analysis was performed for the primary outcome (pregnancy) using Epi Info™ statistical software. Discontinuation rate was defined as the proportion of women who did not continue use of the allocated method to follow-up, out of the women initiating the allocated method and this analysis was, thus, per protocol.

Baseline characteristics were compared between enrolled groups, as well as those with follow up of 12 months or more. For categorical variables, the rates of outcome events were compared as risk ratios (RR) with 95 % confidence intervals (CI). *P*-values were calculated using the Chi-squared test with Mantel-Haenszel correction or, for small numbers, the Fisher’s exact test. *P* values of less than 0.05 were regarded as statistically significant.

### Trial registration

Registration of the protocol with the South African National Clinical Trials Register was undertaken on 15 September 2008 (Verification Code: 0–953). It was subsequently found not to have been logged by the system, and was re-registered with the Pan African Clinical Trials Registry on 4th September 2014 (PACTR201409000880157). (http://apps.who.int/trialsearch/Trial2.aspx?TrialID=PACTR201409000880157).

## Results

The trial closed early due to international concerns regarding a possible association between DMPA and HIV acquisition, following publication of an observational study reporting increased HIV acquisition among women who chose DMPA [7], and the subsequent plan by international stakeholders to conduct a large, multicentre trial that could address the DMPA/HIV question [8].

**Fig. 1:**
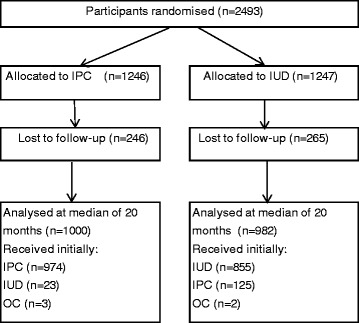
CONSORT diagram showing the flow of participants

At closure, 2,493 of the target sample had accrued (36 %), with 1,247 and 1,246 women randomised to the IUD and IPC groups, respectively. An additional 19 randomisation envelopes could not be linked to participants (see CONSORT Fig. 1). Baseline data for the IUD and IPC groups were well matched (Table 1). Data on the primary outcome were available for 1,963 participants (78.7 %). The median time to final interview was 20 months (interquartile range 15 to 28). In the IUD group, 56 out of 971 women (5.8 %) became pregnant during follow-up compared with 83 out of 992 women (8.4 %) in the IPC group (risk ratio (RR) 0.69, 95 % confidence interval (CI) 0.50 to 0.96; *P* = 0.025). There were no statistically significant differences between groups for the type of pregnancy outcome (Table 2).

**Table 1:** Baseline data expressed as numbers (%) or mean values (standard deviation (SD))

	IUD group		IPC group	
	*n* (%) or mean [SD]	*N*	*n* (%) or mean [SD]	*N*
Age in years	26.7 [6.3]	1234	26.4 [6.1]	1239
Weight in kgs	71.7 [17.9]	1214	70.2 [16.4]	1220
Previous miscarriage	107 (8.7)	1235	100 (8.1)	1232
Previous caesarean section	163 (13.3)	1230	146 (11.8)	1236
Previous pelvic sepsis	241 19.6)	1230	237 (19.2)	1232
Hypertension	14 (1.1)	1236	11 (0.9)	1242
Diabetes mellitus	5 (0.4)	1236	6 (0.5)	1241
HIV positive	236 (21.2)	1110	240 (21.6)	1118
*Previous contraception use*				
-IPC	801 (71.1)	1126	824 (72.5)	1136
-oral contraceptive pill	90 (7.7)	1176	64 (5.4)	1184
-IUD	2 (0.2)	1177	1 (0.1)	1188

*IUD* intrauterine device, *IPC* injectable progestin contraception

**Table 2:** Pregnancy rates and outcomes

	IUD group (*N* = 971)		IPC group (*N* = 992)		RR	95 % CI	*P*-value (M-H)
	*n*	%	*n*	%			
Pregnancy (total)	56	5.8	83	8.4	0.69	0.50 to 0.96	0.025
*Pregnancy outcome*:							
Birth	29	3	41	4.1	0.72	0.45 to 1.15	0.17
Miscarriage	3	0.3	3	0.3			
Termination	2	0.2	2	0.2			
Ectopic	0	0	1	0.1			
Ongoing	16	1.6	29	2.9	0.56	0.31 to 1.03	0.059
Unknown	6	0.6	7	0.7			

*IUD* intrauterine device, *IPC* injectable progestin contraception, *RR* risk ratio, *CI* confidence interval, *M*-*H* mantel-haenszel chi square 2-tail

Data on discontinuation were available for 982 women (78.7 %) in the IUD group and 1000 women (80.3 %) in the IPC group. In the IUD group, 855 (87.1 %) of the 982 women received an IUD as allocated; the other 127 women (12.9 %) received a non-allocated method (protocol violations), which was an IPC in most (125) cases. In the IPC group, 974 out of 1000 women (97.4 %) received an IPC as allocated, with 85.5 % receiving DMPA; the remainder received NET. Twenty-three out of the 26 women in the IPC group receiving a non-allocated method (protocol violations) received an IUD, and follow-up data did not distinguish between DMPA and NET. At the final follow-up interview, 729 women (74.2 %) out of 982 women in the IUD group were using an IUD, compared with 831 (83.1 %) out of 1000 women in the IPC group (intention-to-treat). Loss to follow-up was relatively high and, in the worse case scenario, if all those lost to follow-up were not using the allocated method, the percentage of women using the allocated method at follow-up in the IUD and IPC groups was 58.5 % and 66.7 %, respectively. However, among women who initiated their allocated treatment as per protocol, discontinuation at a median follow-up of 20 months was not statistically significantly difference between the IUD and the IPC groups (141/855 women [16.5 %] versus 143/974 women [14.7 %], respectively; RR 1.12, 95 % CI 0.91 to 1.39; *P* = 0.29) (Table 3).

**Table 3:** Allocated and actual contraception method and discontinuation

	Allocated method					
	IUD			IPC		
Actual method/s used	*N*	% final method known	% total	*N*	% final method known	% total
Initial method used: IUD	855	87.1 %	68.6 %	23	2.3 %	1.8 %
*Initial method*/*final method*:						
IUD/IUD	714	72.7 %	0.1 %	21	2.1 %	1.7 %
IUD/IPC	32	3.3 %	2.6 %	0	0.0 %	0.0 %
IUD/OC	4	0.4 %	0.3 %	0	0.0 %	0.0 %
IUD/Sterilisation	1	0.1 %	0.1 %	0	0.0 %	0.0 %
IUD/none	104	10.6 %	8.3 %	2		0.2 %
Initial method used: IPC	125	12.7 %	10.0 %	974	97.4 %	78.2 %
IPC/IPC	105	10.7 %	8.4 %	831	83.1 %	66.7 %
IPC/IUD	15	1.5 %	1.2 %	11	1.1 %	0.9 %
IPC/OC	0	0.0 %	0.0 %	11	1.1 %	0.9 %
IPC/sterilisation	0	0.0 %	0.0 %	3	0.3 %	0.2 %
IPC/none	5	0.5 %	0.4 %	118	11.8 %	9.5 %
Initial method used: Other	2	0.2 %	0.2 %	3	0.3 %	0.2 %
*Initial method*/*final method*:						
OC/OC	2	0.2 %	0.2 %	3	0.3 %	0.2 %
Final method known	982		78.7 %	1000		80.3 %
Final method unknown	265		21.3 %	246		19.7 %
Total randomised	1247			1246		

*IUD* intrauterine device, *IPC* injectable progestin contraception

Women in the IUD group were more likely than those in the IPC group to discontinue contraceptive use due to abdominal pain or backache (*P* = 0.00005) and non-specific symptoms (*P* = 0.004), and those in the IPC group were more likely to discontinue due to oligo-or amenorhoea (*P* = 0.004) and lack of sexual activity (*P* = 0.0002) (Table 4). Expulsion of the IUD was given as the reason for discontinuation in 26/135 (19.3 %) women who discontinued IUD use, giving an expulsion rate of 3 %. Two women in the IUD group and four women in the IPC group were reported to have died during the follow-up period. Information on these women is shown in Table 5.

**Table 4:** Discontinuation and reasons

Reason for discontinuation (total)	IUD group		IPC group		RR	95 % CI	*P*-value (M-H)
		135		147			
Want baby/married	19	14.1	26	17.7	0.8	0.46 to 1.37	0.41
Fell pregnant	3	2.2	1	0.7			
Abdominal pain/backache	21	15.6	3	2	7.62	2.33 to 25	0.00005
Heavy menstruation	9	6.7	13	8.8	0.75	0.33 to 1.71	0.5
Scanty/no menstruation	0	0	9	6.1	0	NE	0.004 (FE)
Not sexually active	11	8.1	36	24.5	0.33	0.18 to 0.63	0.0002
Weight gain	0	0	3	2			
Weight loss	1	0.7	0	0			
Partner request	1	0.7	0	0			
Discomfort during intercourse	1	0.7	0	0			
Infection	2	1.5	0	0			
Itchy vulvae/discharge	2	1.5	0	0			
Headaches	0	0	1	0.7			
Non-specific symptoms	12	8.9	2	1.4	6.5	1.49 to 29	0.004
Using condoms	1	0.7	4	2.7			
Suspected cancer	1	0.7	0	0			
No reason given	25	18.5	49	33.3	0.53	0.35 to 0.82	0.003
IUD came out	26	19.3	0	0			

*IUD* intrauterine device, *IPC* injectable progestin contraception, *RR* risk ratio, *CI* confidence interval, *M*-*H* mantel-haenszel chi square 2-tail

**Table 5:** Women reported to have died

Age (y)	Baseline HIV status	Weight (Kg)	Group allocation	Option received	Date enrolled	Approximate date of death	Reported cause of death
31	Unknown	81	IUD	IUD	02-Sep-09	2012	Motor vehicle accident
24	Unknown	65	IUD	IUD	24-Feb-10	15-May-10	Motor vehicle accident
18	Negative	67	IP	DMPA	13-Jul-10	Dec-11	Eclampsia
32	Positive	?	IP	DMPA	08-Nov-10	18-Dec-11	Knife wound/s
26	Positive	49	IP	DMPA	10-Nov-11	10-Feb-12	HIV-related infection
20	Negative	60	IP	NET	07-Aug-12	May-13	Asthmatic attack

## Discussion

As mentioned above, during the conduct of our trial an observational study published findings of increased HIV acquisition among women who chose DMPA contraception, which gained wide media attention [7]. Trial investigators attended international stakeholder consultations, where there were calls for a large, multicentre trial to conclusively answer the questions on the effects of hormonal contraception on HIV acquisition and disease progression [2]. In such a trial, any evidence relating to DMPA should be robust and distinct from other long-acting, progestin contraceptives. As the current pragmatic trial was unable to unequivocally answer the DMPA/HIV question due to the allowance of DMPA or NET in the IPC group, we stopped recruitment in November 2012. We followed up the reduced sample as planned with the aim of using these data to inform the design of the requisite, more robust trial [8].

Nevertheless, the sample size was adequate to assess the primary outcome due to the occurrence of higher than expected pregnancy rates in this trial. In contraceptive guidelines, contraceptive failure with typical use is considered to be about six pregnancies per 100 women-years with IPC use, and one per 100 women-years with IUD use [9]. The estimate for IPC failure is supported by our findings, and those of a recent observational study of contraceptive use in South Africa, which reported pregnancy rates with IPC of 4.4 per 100 women-years [3]. However, pregnancy rates in women allocated to IUD use in our study, although significantly lower with the IUD than IPC, were higher than expected. This was probably partly due to over-estimation on intention-to-treat analysis, as 13 % of women allocated to the IUD group received IPC. Thus, our findings provide robust evidence that allocation to the IUD is more effective than allocation to IPC in preventing unintended pregnancies in South Africa. This has important implications for contraceptive policy in South Africa, where injectable progestin contraception accounts for the vast majority of contraception in the public sector and efforts to improve the method mix have had limited success.

A large study of more than 200,000 women from 14 developing countries reported a median probability of IUD discontinuation of 13.2 % [10]. Higher rates of IUD and IPC discontinuation (and unintended pregnancy) have been observed among women with a history of termination of pregnancy [11–14]. In two post-termination cohorts of women who chose IPC, discontinuation rates as high as 64 per 100 woman-years, and 84 % were reported [13, 14]. As all the participants in our trial were recruited post-termination of pregnancy, this factor may have contributed to contraceptive failure and discontinuation in our trial.

Due to the pragmatic nature of this trial, loss to follow-up was relatively high, at around 20 % in both intervention groups. This limitation could have lead to an under-estimation of method discontinuation due to differential loss of women who discontinued their allocated method. For this reason we reported the proportion of women using each method out of those with known follow up data, as well as for of the intervention groups as a whole. Baseline characteristics were comparable, therefore, loss to follow-up did not appear to have been differential between random allocation groups. Another limitation of the trial was the high number of protocol violations in the IUD group; 12.7 % of women with follow-up data in the IUD group received the IPC after randomisation instead of the IUD. The reasons for protocol violations were not recorded, but could have been due to women declining the IUD after randomisation in favour of the more familiar method (IPC), or difficulties with IUD insertion, or provider barriers. Of the participants with data on previous contraceptive use, 1625 had previously used IPC versus only three previously using the IUD (Table 3). Whilst protocol violations may have underestimated differences between contraceptive methods on pregnancy rates, in intention-to-treat analysis they are unlikely to have over-estimated effect differences.

While individual choice is a cornerstone of contraceptive services, this trial demonstrates that after counseling on different contraceptive options many women did not have a specific preference for a contraceptive method and were willing to agree to randomisation without any financial remuneration or other benefits. Although initial compliance in the IUD group was sub-optimal (87 %), the acceptability of randomisation is supported by the fact that discontinuation of allocated methods were comparable with observational studies in which women had chosen their contraceptive method [10–14]. No trial-related steps were taken to support adherence to the allocated contraception method, and no payments or other incentives were offered, other than payment of transport costs for the final visit at 12 or more months post-randomisation. In addition, the provision and follow up of the allocated contraceptive method took place within the routine health service. The advantage of this was that relative discontinuation rates and contraceptive effectiveness were more likely to reflect the ‘typical-use’ effect of these methods in routine practice (though absolute rates might differ from women choosing a specific method). The disadvantage was that follow-up was less efficient and protocol violations were higher than might have been achieved with a more intensive research intervention.

## Conclusions

The IUD was more effective than IPC in a post-termination of pregnancy setting in South Africa. Disontinuation rates with the IUD were comparable to the more familiar and widely accepted IPC, suggesting that efforts to expand contraception method choice and overcome user and provider barriers to IUDs are worthwhile. Study limitations such as protocol violations may have led to an under-estimation of differences in the primary outcome, but over-estimation is unlikely. This research supports the feasibility of conducting a large, multi-centre randomised trial to address the DMPA/HIV question.
